# Refining estimates of availability bias to improve assessments of the conservation status of an endangered dolphin

**DOI:** 10.1371/journal.pone.0194213

**Published:** 2018-03-13

**Authors:** Federico Sucunza, Daniel Danilewicz, Marta Cremer, Artur Andriolo, Alexandre N. Zerbini

**Affiliations:** 1 Laboratório de Ecologia Comportamental e Bioacústica, Programa de Pós-graduação em Ecologia, Universidade Federal de Juiz de Fora, Juiz de Fora, Minas Gerais, Brazil; 2 Instituto Aqualie, Juiz de Fora, Minas Gerais, Brazil; 3 Grupo de Estudos de Mamíferos Aquáticos do Rio Grande do Sul, Torres, Rio Grande do Sul, Brazil; 4 Universidade Estadual de Santa Cruz, Ilheus, Bahia, Brazil; 5 Laboratório de Nectologia, Departamento de Ciências Biológicas, Universidade da Região de Joinville, São Francisco do Sul, Santa Catarina, Brazil; 6 Marine Mammal Laboratory, Alaska Fisheries Science Center, National Oceanic and Atmospheric Administration, Seattle, Washington, United States of America; 7 Cascadia Research Collective, Olympia, Washington, United States of America; 8 Marine Ecology and Telemetry Research, Seabeck, Washington, United States of America; Sanya Institute of Deep-sea Science and Engineering Chinese Academy of Sciences, CHINA

## Abstract

Estimation of visibility bias is critical to accurately compute abundance of wild populations. The franciscana, *Pontoporia blainvillei*, is considered the most threatened small cetacean in the southwestern Atlantic Ocean. Aerial surveys are considered the most effective method to estimate abundance of this species, but many existing estimates have been considered unreliable because they lack proper estimation of correction factors for visibility bias. In this study, helicopter surveys were conducted to determine surfacing-diving intervals of franciscanas and to estimate availability for aerial platforms. Fifteen hours were flown and 101 groups of 1 to 7 franciscanas were monitored, resulting in a sample of 248 surface-dive cycles. The mean surfacing interval and diving interval times were 16.10 seconds (SE = 9.74) and 39.77 seconds (SE = 29.06), respectively. Availability was estimated at 0.39 (SE = 0.01), a value 16–46% greater than estimates computed from diving parameters obtained from boats or from land. Generalized mixed-effects models were used to investigate the influence of biological and environmental predictors on the proportion of time franciscana groups are visually available to be seen from an aerial platform. These models revealed that group size was the main factor influencing the proportion at surface. The use of negatively biased estimates of availability results in overestimation of abundance, leads to overly optimistic assessments of extinction probabilities and to potentially ineffective management actions. This study demonstrates that estimates of availability must be computed from suitable platforms to ensure proper conservation decisions are implemented to protect threatened species such as the franciscana.

## Introduction

Estimates of abundance are fundamental to properly assess the conservation status and to effectively design and implement management actions of threatened species or populations [1]. However, field-based research is commonly plagued by imperfect detection of the subjects of interest, often resulting in biased estimates of population parameters [2, 3]. For many marine species (e.g., cetaceans and chelonians) line transect aerial surveys constitute one of the most effective methods to estimate abundance [4]. In order to compute unbiased estimates, line transect methods assumes that detection on the survey trackline is certain (i.e., *g*(0) = 1) [2], but in aerial surveys visibility bias (i.e., *g*(0) < 1) often leads to underestimation of animals abundance [5, 6]. Marsh and Sinclair [7] coined the terms perception and availability bias to differentiate two forms of visibility bias during aerial surveys. Perception bias occurs when animals are available to be seen but are missed by the observers while availability bias correspond to animals that are missed because they are submerged and unavailable.

Availability bias can be a substantial source of bias for aerial surveys of marine mammals [5, 8, 9]. The high speed of the aircrafts reduces the amount of time a given area of the ocean is within the observer’s detectable view, resulting in a high proportion of undetected animals in this type of surveys. For example, Laake et al. [5] estimated that as many as 70% of harbor porpoises (*Phocoena phocoena*) would not be available to observers in an fixed-wing aircraft during aerial surveys in coastal waters of Washington State (USA). Accounting for this source of bias has, therefore, great relevance for population estimates, particularly of threatened species.

The franciscana, *Pontoporia blainvillei*, is a small dolphin endemic to coastal waters in the western South Atlantic Ocean. It occurs in waters typically shallower than 30m [10] from Itaúnas, Brazil (18º 25’S) to Golfo San Matías, Argentina (41º 10’S) [11, 12]. The species is considered the most threatened small cetacean in South America due to high, and possibly unsustainable, bycatch levels as well as increasing habitat degradation [13]. Incidental catches in fishing gear have been reported along most of the species’ range for almost seventy years [13–15]. In order to guide conservation and management actions at a regional basis, the franciscana range was divided into four zones known as 'Franciscana Management Areas'—FMAs: two in southeastern and southern Brazil (FMA I and II), one in southern Brazil and Uruguay (FMA III) and one in Argentina (FMA IV) [16, 17]. The franciscana is currently listed as Vulnerable by the IUCN Red List of Threatened Species [18] and Critically Endangered by the Brazilian Government [19].

Because the franciscana is typically difficult to see in the wild [20], computing abundance estimates of this species has long been regarded as a challenge for conservation biologists [21, 22]. Line transect aerial surveys have been considered the most efficient method to estimate abundance of this species [23] and have been applied across almost their entire range [22, 24–26]. Early estimates of franciscana abundance from aerial surveys [22, 24, 25] have been considered unreliable because, among other issues, they did not properly accounted for visibility bias [25, 27]. Dual platform experiments showed that franciscana density estimates from boat surveys are nearly 4–5 times greater than those obtained with aerial surveys, demonstrating that visibility bias (as well as underestimation of group sizes) represents a significant problem in aerial surveys for the species [28]. However, because this experiment was unable to separate the contribution of perception and availability bias to total visibility bias, further research was needed to better understand how these two sources of bias influence estimates of abundance.

To date, estimates of availability bias used to correct franciscana aerial surveys were computed from dive parameters collected from boats or land-based sites [24, 29]; and therefore may not properly reflect the amount of time franciscana groups are available to observers conducting aircraft-based surveys [25]. The main objective of this study is to estimate correction factors for availability bias using aerial platforms. In particular, helicopter surveys were used to estimate surface and dive intervals of franciscana groups as perceived from the air in order to (1) evaluate potential biological and environmental variables influencing dive parameters and (2) assess the magnitude of the differences in availability computed using data from surface-based and aerial platforms.

## Methods

### Study area

**Fig 1:**
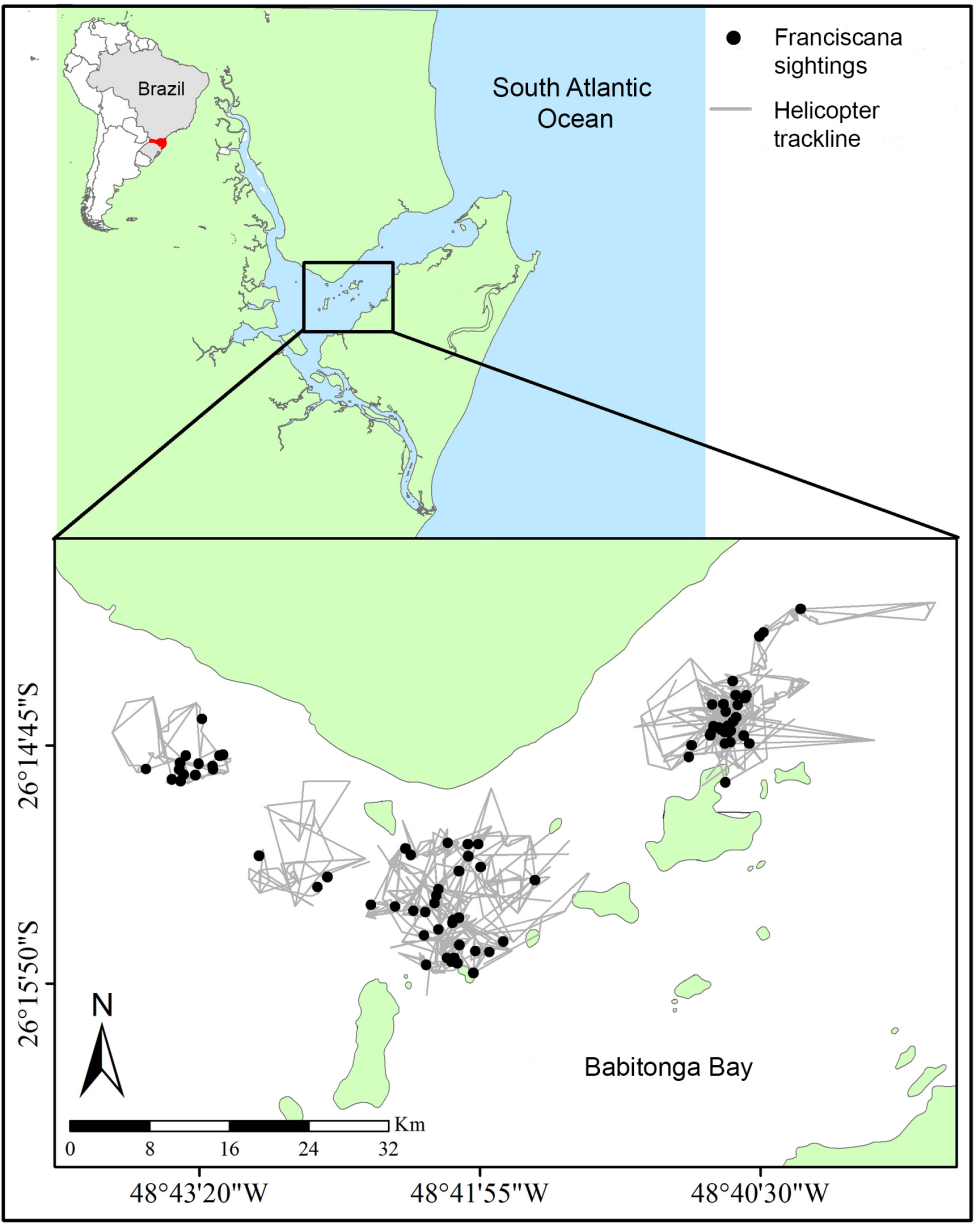
Study area. Map of Babitonga Bay, State of Santa Catarina (red), southern Brazil. The inset shows the realized trackline effort and franciscana (*Pontoporia blainvillei*) sightings from helicopter surveys.

Aerial surveys were conducted in Babitonga Bay (26º 16’S, 048º 42’W), state of Santa Catarina, southern Brazil from 23 to 31 January 2014 (Fig 1). There is a number of advantages in conducting this study in this region. First, in Babitonga Bay, franciscanas predictably occur in relatively large densities throughout the year and show limited or no avoidance to small boats [30]. Second, group sizes seen in that region (mean = 2.90, range = 1 to 7 individuals, [28]) are representative of those seen throughout the range of the species (e.g., [11, 29]). Finally, the bay is relatively protected and therefore provides good weather conditions (e.g., relatively calm waters) for sighting surveys. In addition, previous studies to understand visibility bias have been conducted in this area [28], favoring a direct comparison of new information with existing data.

### Data collection

Visual surveys were conducted from a Robinson R44 four-seat helicopter at 500 ft (150 m), an altitude consistent with that flown during aerial surveys to estimate abundance of this species (e.g., [25, 28]). Surveys had an average duration of 4 h and were carried out during the morning, when wind conditions were calm, optimal for this type of survey. The doors of the helicopter were detached during the study to maximize visibility for the observers. Two scientists with substantial experience in aerial surveys and familiar with the identification of franciscanas searched for groups of dolphins on the left side of the helicopter. Once a group was detected, the pilot hovered over it and each observer recorded surfacing and dive times independently. A group was defined as an aggregation of dolphins in close proximity of each other, moving in the same direction and in apparent association [31]. Visual and acoustic communication between the two observers were used to minimize simultaneous recording of the same group.

A record of sequences of surface-dive cycles started at the moment a group that had been detected became unavailable (dove), immediately after first being sighted, and ended when the observer lost the group. The first post-sighting interval at the surface was discarded from the analysis because it was not possible to ensure that the group was detected immediately after it became available. Inclusion of the first post-sighting interval could lead to underestimation of the surfacing interval and, consequently, to biased estimates of the length of the surface-dive cycle. Because the perspective from the helicopter makes it difficult distinguish individual franciscanas, observers recorded surfacings of any animal in the group. For each sampling period, the following information was registered: i) number of individuals visually available at, or near, the surface, ii) group size, iii) presence of calves and iv) a subjective assessment (categorized as: high, moderate, and low) of the level of confidence the observers had that they tracked the same group while collecting surfacing and dive times. In addition, the observers recorded *ad libitum* behavioral observations [32]. Based on the experience of the observers, "unusual" behaviors like an abrupt dive, a sudden turn away from the helicopter or an increase in swimming speed, were subjectively classified as a disturbance response to the presence of the helicopter.

Environmental conditions, including sea state (Beaufort scale), intensity and angle of glare, water color, and an assessment of the overall visibility conditions were recorded at the beginning and end of each sample or when the conditions changed. Data were recorded on audio digital recorders and time was synchronized to a GPS to allow for geo-referencing of each observation. Depth and water transparency (measured with a Secchi disc) at the location of each sighting were also recorded from boats operating in the same area and in radio communication with the helicopter. Tide level was obtained from the tide chart of the Port of São Francisco do Sul (26º 14’S, 048º 37’W).

### Data analysis

#### Definition of dive parameters

A surfacing interval was defined as the period of time in which at least one franciscana in the group was visually available to the observers in the helicopter at or near the surface. A diving interval was defined as the period of time in which all individuals of the group were not visible to the observers in the helicopter. A surface-dive cycle was defined as the period of time from the beginning of one surfacing to the next [6]. The proportion of time franciscana groups were visually available for detection from an aerial platform, hereafter referred to as “proportion at surface”, was computed as *s*_*i*_/*s*_*i*_+*d*_*i*_, where *s*_*i*_ and *d*_*i*_ correspond, respectively, to the surfacing interval and diving interval times of the surface-dive cycle *i* (*i* = 1, 2,…, *n* surface-dive cycles).

#### Mixed-effects models

Generalized linear mixed-effects models (GLMMs) [33,34] were used to evaluate the effects of biological and environmental predictors on the proportion at surface (the response variable) using package nlme [35] in the open source software R [36]. Because the same group may have been resampled during records of surface-dive cycles, individual franciscana groups were fitted as a random effect. The full set of models included all possible permutations of five predictor variables, which potentially affect the proportion at surface. This potential effect is interpreted here as the influence of the predictor variable on the behavior of the dolphins and/or the observers' ability to detect an individual. "Group size" describe the total number of individuals in the group. Because sample sizes were insufficient to fit models for all group size values, this variable was grouped into two categories *small* (groups with 4 or less individuals) and *large* (groups with more than 4 individuals). "Presence of calves" indicate the presence or absence of calves in the group. "Depth" and “Water Transparency” describe, respectively, the depth of the water column and the transparency at the location where the group was detected. "Tidal level" represents the level of the tide when surface-dive cycles were recorded. The proportion at surface was log-transformed to reduce the heterogeneity of the variance and to approximate the response variable to a normal distribution. Because multiple surface-dive cycles recorded for each franciscana group were temporally and spatial correlated, consideration was given to include an autocorrelation structure in the mixed effects models. Global models with (*g*1) and without (*g*2) autocorrelation structures were compared using the Akaike's Information Criterion AIC. Correlation structures of order 1 (AR-1) or an autocorrelation-moving average of order (*p*,*q*) were considered. The tested number of autoregressive parameters (*p*) and moving average parameters (*q*) varied between 1–2 and 0–1, respectively. To test whether the most supported autocorrelation structure, i.e., autocorrelation structure of order 1 (AR-1), was needed, the global model with (*g*1) and without (*g*2) the AR-1 structure were compared using AIC. Because the inclusion of the AR-1 structure resulted in a considerable improvement in AIC (*g*1 AIC = 515.792, *g*2 AIC = 520.344), all proposed models included the AR-1 structure.

The GLMM model selection was based on AIC, and the model with the smallest AIC value was considered the most parsimonious approximating model within the full set of models [37]. Akaike weights *w*_*i*_ were calculated for each model as a representation of the probability of the model be the actual “best model” within the full set of models [37]. Inference about the relative importance (RI) of each predictor variable in determining the proportion at surface was based on the sum of Akaike weights of each variable across all candidate models containing the variable, and ranged from 1 (most important) to 0 (least important). Model averaging was conducted across the full set of models, and model-averaged parameters were estimated for each predictor variable, with unconditional standard errors incorporating model uncertainty [37]. Model averaging was performed using the package MuMIn [38].

#### Estimation of availability

To estimate the probability of one franciscana group be visually available within the visual range of a passing observer in a fixed-wing aircraft, or availability (*Pr*) of franciscana groups, the method proposed by Laake et al. [5] was used:

$$Pr=\frac{E(s)}{E(s)+E(d)}+\frac{E(d)[1-e^{-\frac{w(x)}{E(d)}}]}{E(s)+E(d)}$$ (1)

where *E*(*s*), *w*(*x*), and *E*(*d*), correspond, respectively, to the mean time of each individual surfacing interval, the window of time during which a franciscana group is in the observer’s view at a distance *x* and the mean time of each individual diving interval. Availability was estimated for all groups together and for *small* and *large* groups.

*w*(*x*) was calculated following the McLaren’s [39] model:

$$w(x)=\frac{\sqrt{r^{2}+x^{2}}}{v}$$ (2)

where *v* is the mean aircraft velocity used in franciscana aerial surveys (*v* = 50 m/s), *r* is the radius of the observer’s view area (*r* = 300 m) and *x* is the distance from the survey line, assumed to correspond to zero for the computation of availability.

Standard errors and confidence intervals were estimated using a nonparametric bootstrap procedure [40]. For each bootstrap replicate, franciscana groups were sampled with replacement to match the original number of franciscana groups recorded (n = 101, see below) during the surveys and an estimate of *Pr* was obtained as in Eq 1. This process was repeated 1,000 times and the mean *Pr* and its standard error were computed from these replicates using standard formulae. Ninety-five percent confidence intervals were estimated by taking the 2.5 and 97.5 percentiles of the bootstrap replicates. Availability bias is equal to the complement of *Pr*.

#### Effects of platform type on the estimation of dive parameters

To assess the effect of using dive parameters recorded from surface-based and aerial platforms to compute availability, the ratios of dive parameters (i.e., *E*(*s*) and *E*(*d*)) recorded in this study relative to those recorded from surface-based platforms [24, 29] was computed. Surface-based observations of franciscanas were obtained from shore-based platforms and boats in Anegada Bay, Argentina (see [29] for methods).

In addition, estimates of availability obtained with dive parameters from surface-based platforms (*Pr*[surface]) and applied to correct for availability bias in previously estimates of franciscana abundance from aerial surveys [22, 24, 26], were recomputed with data provided in this study (*Pr*[aerial]). For comparison, the same values and analytical methods as presented in the original studies were used. Secchi et al. [22] used dive parameters from Bordino et al. [29] and studies by Crespo et al. [24] and Zerbini et al. [26] used dive parameters from Crespo et al. [24]. Availability in all these studies was calculated with the method proposed by Barlow et al. [41].

## Results

**Fig 2:**
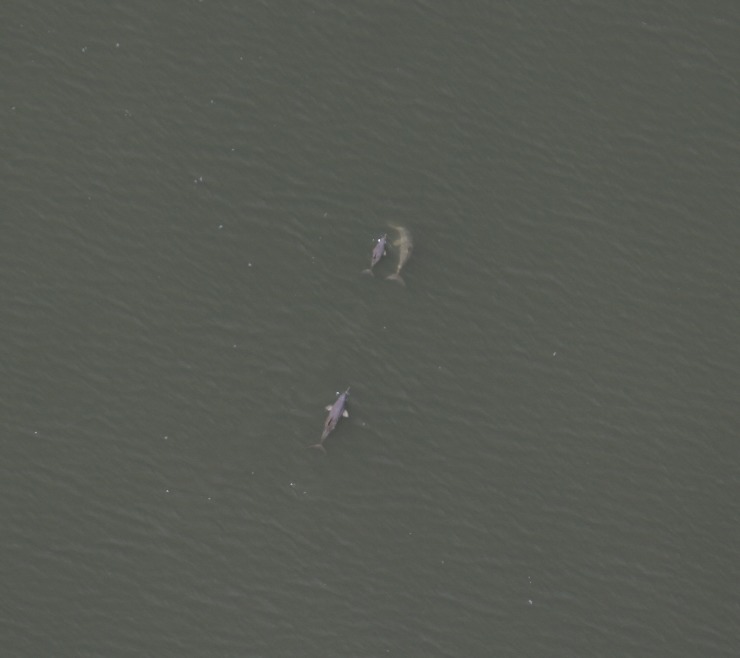
Franciscana (*Pontoporia blainvillei*) group. Group classified as available to detection from the air.

A total of 15 hours were flown during this experiment. Dolphins were well defined as visually available at some depth beneath the surface (Fig 2). A total of 337 complete surface-dive cycles was recorded for 120 franciscana groups in the original dataset. Franciscana groups appeared not to react to the presence of the helicopter very often. In only 2% (n = 6) of all recorded surface-dive cycles a response (e.g., an abrupt dive) was observed. After the removal of cycles were a reaction was documented as well as those classified as moderate/low confidence, the dataset for analysis was reduced to 248 complete surface-dive cycles from a total of 101 groups (mean = 2.45 cycles/group, SE = 1.99, range = 1–10). Biological and environmental variables recorded during this study are summarized in Table 1.

**Table 1:** Summary of biological and environmental variables recorded in this study and tested in the generalized mixed-effects models.

Variable	Factor/Numeric	Levels	Mean	SE
Group size	Factor	*small* (1–4) and *large* (5–7)	2.92	1.11	
Presence of calves	Factor	*yes* and *no*	0.26*	NA	
Water transparency (cm)	Numeric	77–162	110.1	23.90	
Depth (m)	Numeric	4.4–12	7.02	1.87	
Tide level (m)	Numeric	0.3–1.2	0.73	0.24	

*Proportion of groups (n = 101) with calves (n = 26)

### Mixed-effects models

The most parsimonious approximating model to assess the influence of biological or environmental variables to the proportion at surface only included group size as the predictor variable (Table 2). This model suggested that time spent at the surface was positively correlated with the size of the group (*p* = 0.004). Group size was the most important predictor variable (RI = 0.96), followed by tidal level (RI = 0.36), water transparency (RI = 0.31), depth (RI = 0.30) and presence of calves (RI = 0.27) (Table 3). Except for group size, the other predictor variables had little influence in the estimated proportion at surface. The response variable slightly increased with increasing tide and depth and decreased with increasing water transparency and with the presence of calves in the group, but the effect of these variables was not statistically significant (Table 3).

**Table 2:** Most supported mixed-effects models (ΔAIC≤ 2) used to assess the influence of biological and environmental variables in the proportion at surface of franciscana groups.

Model	AIC	ΔAIC	*w*_*i*_
Group size	482.85	0.00	0.22
Group size + Tidal level	483.99	1.14	0.12
Group size + Water transparency	484.51	1.66	0.10
Group size + Depth	484.72	1.87	0.09
Group size + Presence of calves	484.83	1.98	0.08

**Table 3:** Model-averaged predictor coefficients and relative importance (RI).

Variable	*β*	SE	RI
Group size (large)	0.44*	0.18	0.96
Tidal level	0.06	0.14	0.36
Water transparency	-0.0003	0.001	0.31
Depth	0.003	0.01	0.30
Presence of calves (yes)	-0.0004	0.05	0.27

*β* = coefficients values for the averaged model

SE = standard error.

*Statistically significant different from zero

### Dive parameters and estimation of availability

**Fig 3:**
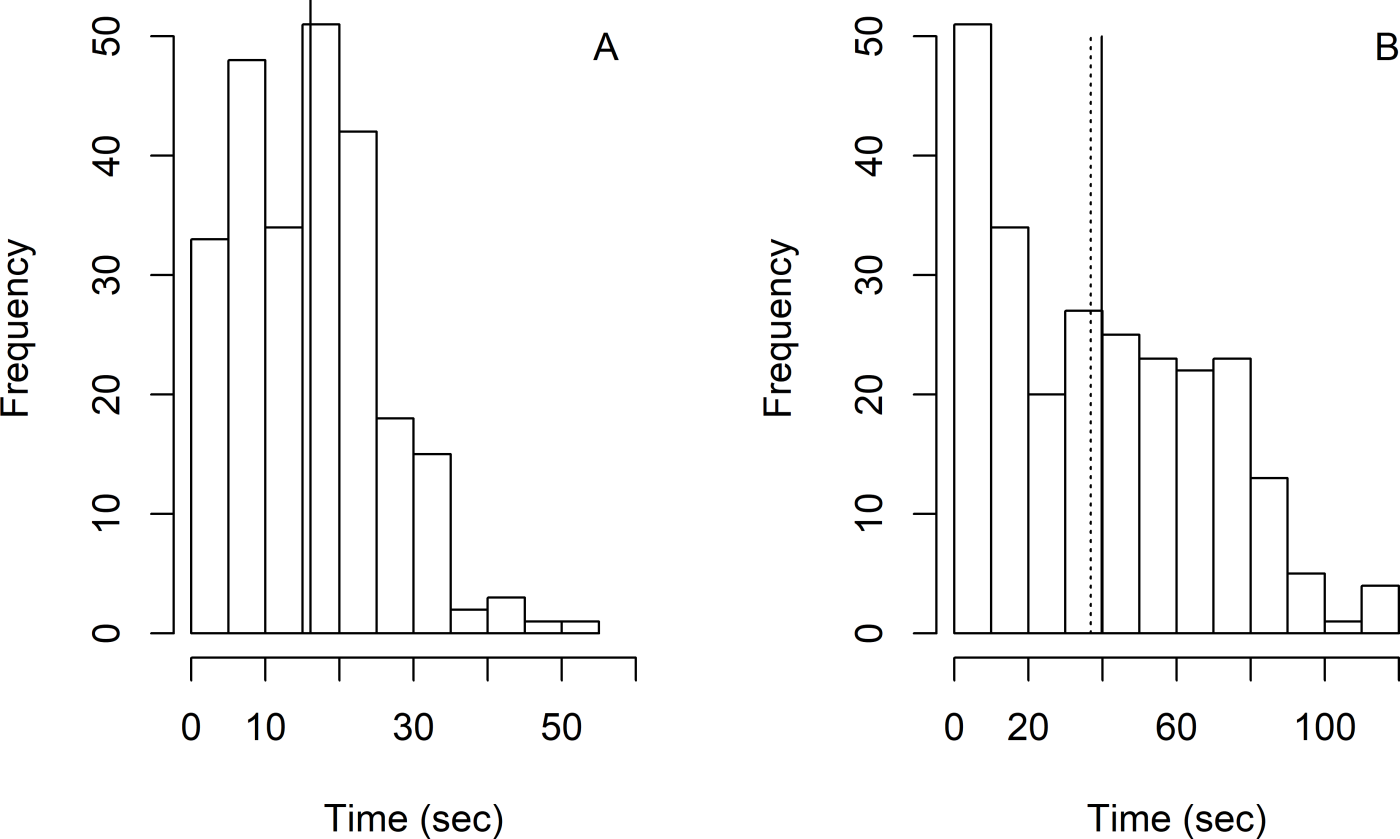
Histograms of surfacing (A, solid line represents the mean and the median) and dive (B, solid line represents the mean, and dashed line the median) times of franciscana (*Pontoporia blainvillei*) groups recorded from helicopter surveys in Babitonga Bay, southern Brazil.

Surfacing intervals varied from 1.03 seconds to 51.74 seconds (median = 16, mean = 16.10, SE = 9.74) (Fig 3A) and diving intervals varied from 0.46 seconds to 114.89 seconds (median = 36.86, mean = 39.77, SE = 29.06) (Fig 3B). The average proportion of time at surface was estimated at 0.36 (median = 0.29, SE = 0.22). The estimated window of time (*w*(*x* = 0)) was 6 seconds, which resulted in an estimation of availability of 0.39 (SE = 0.01) for all group sizes combined. Availability of large groups was significantly greater than that of small groups (Table 4).

**Table 4:** Availability of franciscana (*Pontoporia blainvillei*) groups estimated for all groups together and for small and large groups separately assuming a window of time (*w*[*x*]) equal to 6 seconds.

Group category	*n*	Availability (SE, 95% CI)
All groups	101	0.39 (0.01, 0.36–0.42)
Small groups (≤ 4 individuals)	92	0.37 (0.01, 0.34–0.40)
Large groups (> 4 individuals)	9	0.66 (0.08, 0.54–0.83)*

Standard error (SE) and 95% confidence intervals (CI) in parenthesis.

*Indicates statistical significance (based on non-overlaping confidence intervals)

### Effects of platform type on the estimation of dive parameters

The mean surface (*E*(*s*)) and dive (*E*(*d*)) times computed from the helicopter were greater than *E*(*s*) and *E*(*d*) computed from surface platforms at Anegada Bay, Argentina (Table 5). As a consequence, the use of dive parameters obtained at the surface level led to negatively biased estimates of availability of franciscanas for observers surveying from an aerial platform. The magnitude of the bias can be quite substantial, reaching as much as 46% depending on which dive parameters were used in the availability estimates (Table 6).

**Table 5:** Mean (SE in parenthesis) surfacing (*E*(*s*)) and diving (*E*(*d*)) times of franciscana groups recorded from different observation platforms (S = surface [land/boat], A = aerial [helicopter]).

Platform	Study Area	*E(s)*	*E(d)*	*E(s)*^A^/ *E(s)*^S^	*E(d)*^A^/ *E(d)*^S^	Source
S	Anegada Bay, Argentina	1.2 (0.4)	21.7 (19.2)	13.42	1.83	Bordino et al. [29]
S	Anegada Bay, Argentina	1.2 (0.4)	27.95 (4.41)	13.42	1.42	Crespo et al. [24]
A	Babitonga bay, Brazil	16.10 (9.74)	39.77 (29.06)	-	-	This study

*E*(*s*)^A^/*E*(*s*)^S^ and *E*(*d*)^A^/*E*(*d*)^S^ represent the ratios of *E*(*s*) and *E*(*d*) recorded from aerial and surface platforms, respectively. Data from Crespo et al. [24] correspond to a compilation of dive parameters presented by Bordino et al. [29, 42].

**Table 6:** Comparison between estimates of availability obtained with dive parameters from land/boat based surveys (Pr[surface], 20, 22, 24) and those computed with data provided in this study (Pr[aerial]) for aerial surveys conducted in three different FMAs.

Region [Study]*	*w*(*x*)	Pr [surface]	Pr [aerial]	Difference
1) FMA II and IV [24, 26]	7.0	0.28 (0.04)	0.41 (0.06)	46%
2) FMA III [22]	7.0	0.36 (0.09)	0.41 (0.06)	14%

*w*(*x*) = window of time.

*Zerbini et al. [26] used the value of Pr [surface] computed by Crespo et al. [24].

## Discussion

Helicopter surveys in Babitonga Bay allowed for successful estimation of availability of franciscana groups. Dolphins were clearly defined as visually available or unavailable, facilitating the unambiguous estimation of the availability process [6]. However, the results presented in this study make the assumption that the helicopter did not influence the diving behavior of the franciscanas apart from those groups for which obvious reactions were observed. Because these responses were so infrequent (just 2% of our sample) and because behavior patterns were similar to those observed from boats in the same area [30, 43] and from the plane during aerial surveys, we conclude that the helicopter had a negligible influence on the behavior of the franciscanas for the majority of the dive cycles measured here. Experiments similar to the one conducted in this study with other species of dolphins of similar total body length of the franciscana (i.e., *Phocoena phocoena* [41] and *Cephalorhyncus hectori* [8]) indicated no evidence that the dolphins were disturbed by the presence of the helicopter supporting our conclusion. If our assumption is violated, however, estimates of dive parameters and availability presented here are biased. Studies (e.g., from surface platforms) comparing behavioral parameters such as time at surface and dive time in the presence and absence of the helicopter could be used to evaluate this assumption further.

Results from the GLMMs indicated that group size was the most important factor influencing the proportion at surface of a franciscana group. Because a surfacing interval was defined as the period of time in which at least one individual in a group was visually available to the observers at, or near, the surface, it is expected that an increase in the group size will increase the likelihood that the group is visually available to be detected. The positive effect of the group size on the availability observed in this study is not surprising and had been previously reported for other species of dolphins (e.g., [8]) and whales (e.g., [44]).

Data from time-depth recorders (TDRs) have been used to correct for availability bias in previous abundance estimates from aerial surveys [9, 45], but the use of these type of data has to be carefully considered depending on the characteristics of the species being surveyed. TDR data may provide information on the probability that the tagged individual is at, or near, the surface and available for detection, but they do not account for the behavior of all individuals in a group (in case animals are seen in groups). Lack of synchronous behavior may be a major concern about the use of data from TDRs to estimate availability because even when the tagged individual is underwater (and potentially unavailable), other individuals in the group may be, making that group detectable. If the size of the group has an effect on the probability of that group being available, such as observed in this study, estimates of availability computed from TDR dive data could misrepresent the true availability process of animals that do not swim in perfect coordination when in a group. Here, the average availability of *small* groups was significantly lower than that of *large* groups. However, the sample of *large* groups was small (n = 9, only about 10% of the total groups recorded), indicating that a more robust evaluation of the group size effect would be appropriate as more experiments are done and larger samples become available.

Use of the TDR data to assess availability of cetaceans can also be compromised by other factors such as water transparency and the characteristics of the body of the animal. In some circumstances, animals with conspicuous features (e.g., large body size and coloration that contrasts well with the water) could be detected from an aerial platform well below they break the surface or after they submerge following a surfacing. In these situations, the TDR data may indicate animals are unavailable when they are still visible. Experiments using TDRs attached to physical models, as the ones proposed by Pollock el at. [9], are important to evaluate these effects.

Another important aspect to consider with respect to availability of groups of different sizes is the need to accurately estimate the number of individuals in the group. Generally, determining the exact number of individuals is difficult and thus group size needs to be estimated. Such estimates are prone to biases from a number of factors including distance from the observer, behavior, body features (e.g., coloration and size), weather or sea conditions, and the size of the group itself (e.g., it is easier to estimate the number of animals in a group of 10 versus a group of 500 individuals). Therefore, before applying correction factors that are specific to group sizes or group size categories, the investigator shall ensure that the number of individuals in a group is accurately estimated during the survey. In this study, we assumed group size were estimated accurately because the helicopter provides an exceptional view to count individuals within the range of group size that franciscanas are observed in Babitonga Bay. Our assumption is further supported by group size estimates independently obtained during boat surveys [28]. The mean size and range of groups (mean = 2.90, SE = 1.24, range = 1–7) estimated by these independent surveys is identical to the ones computed from the helicopter.

Some of the environmental predictors examined in this study have been demonstrated to affect availability of marine mammals (e.g., water transparency, depth—[9, 46]). However, in the present experiment, they were neither included in the most parsimonious approximating model nor they seemed to influence the estimation of availability to a large extent. This can be explained, at least partially, by the relatively narrow range of the values recorded for some of these covariates in this study (e.g., water depth only varied between 4.5 and 12m). These ranges were limited because Babitonga Bay is a relatively confined environment and/or because the survey was conducted in a relatively short period of time (e.g, in the case of water transparency as it is expected to change during dry and rainy seasons). While many of the covariates analyzed in this study are representative of much of the habitat throughout the range of the franciscana, they may not have been sufficient to influence the behavior of the dolphins and/or the observers' capacity to detect an individual.

The effect of some environmental variables (e.g., Beaufort sea state) on estimates of the availability of marine mammals can be minimized by restricting the surveys to optimal survey conditions, however some variables such as water transparency and depth can vary across the species habitat and can change within few minutes of aerial survey time [9] (e.g., as the sampling platform moves between inshore and offshore habitats). This is the case for the franciscana, a species inhabiting habitats with very turbid waters near river mouths as well as clearer waters in the open ocean [24]. While the estimates of availability provided here represent a significant progress towards computing more reliable correction factors for estimates of abundance based on aerial surveys, it is important to recognize that their use could lead to bias in abundance if availability varies significantly across these habitats [47]. In this sense, experiments similar to the one conducted here are recommended for other habitats along the range of the franciscana where environmental characteristics differ from those at Babitonga Bay. These experiments would contribute to better understand the influence of spatially and temporally-varying environmental predictors on the availability of franciscanas to aerial platforms and lead to more robust estimates of availability.

Account for biological and/or environmental-specific availability correction factors in abundance estimates is challenging and highly dependent on proper field methods and robust survey designs. Dynamic environmental variables that can change dramatically while flying (e.g., water transparency) as well as factors that require estimation (e.g., group size), are of great concern when availability correction for different levels of these covariates is expected and when fine scale data on the variability of these covariates are not available. However, fine-scale correction factors may not be realistic on many surveys, and a lack of fine scale measures can be accommodated depending principally on the spatial extend of the survey [47]. If the survey area can be stratified in a way that availability vary among strata, but remain relatively uniform within it, broad categories of stratification could result in substantial improvement on estimates [47, 48]. Abundance estimates corrected for biological and/or environmental-specific availability estimates can be computed using the methods proposed by Pollock et al. [9].

A comparison of the dive parameters sampled from a helicopter in Babitonga Bay [this study] with those obtained from surface platforms in Anegada Bay, Argentina [24, 29] highlights the importance of collecting dive data from aerial platforms in order to develop appropriate availability correction factors for estimates of abundance computed from aerial surveys. Although regional differences in the behavior of franciscanas and in the environmental characteristics between the two areas could influence these results, it is clear that animals not only become available to observers in the air for some time before they break the surface, but also remain visible between short dives [8, 41, this study]. Therefore, the surfacing interval estimated by platforms at the water level (e.g., land or a boat) is expected to be much shorter. In fact, results of this study showed that the surfacing interval recorded from the helicopter were, on average, 13 times greater than the surfacing time recorded from a surface-based platform. The use of surfacing-diving intervals computed from a surface platform may result in (often substantially) negatively biased estimates of availability for aerial platforms. More important, correction factors developed from such estimates of availability would lead to an overestimation of abundance in aerial surveys.

Estimates of franciscana abundance to data have been computed for all FMAs [22, 24–26, 49]. Those for FMA II [26], FMA III [22, 25] and FMA IV [24] have been corrected for availability bias using correction factors computed from the surface-dive intervals obtained through surface platforms. Because the results of this study show that these correction factors were negative biased (by as much as 46%), re-computed abundance estimates for these regions is appropriate, taking the availability estimates provided here into account.

Recalculations are not attempted in this study for the estimates from the FMA III [22, 25] or the FMA IV [24] because these did not account for perception bias and are therefore biased irrespective of how correction for availability is calculated. On the other hand, a re-evaluation of the estimate for the FMA II [26] is possible because an independent estimate of perception bias is available. The estimate originally provided for this region in 2008 (N = 8,525, CV = 0.34) would be reduced by 38% (N = 6,146, CV = 0.35) when availability computed with surface-dive cycles sampled from the aerial platform is used. The estimation of bycatch removals illustrates the management implications of this bias for the franciscanas in FMA II. The estimated bycatch in this area ranged between 300 and 500 individuals per year in the 2000s [14, 27, 50]. This represented an annual removal of 3.5–5.8% of the population as originally estimated by Zerbini et al. [26]. However, removal was substantially larger (4.8–8.6%/year) when calculated using the estimate corrected for availability computed with surface-dive data from the aerial platform, clearly indicating a significantly greater conservation problem.

## Conclusions

The results presented here show that availability bias can account for a large proportion of missing animals during aerial surveys (nearly 60%). In addition, they demonstrate that estimation of availability correction factors for aerial surveys should be computed with data collected from aerial platforms, not from land or boats. Although the results provided here can be refined, they represent a substantial improvement on availability correction factors previously used for franciscana aerial surveys [22, 24–26]. The conservation of the franciscana is a matter of great concern. The species is listed as vulnerable by the IUCN [18] and mortality resulting from incidental catches is thought to be unsustainable through its range [13, 14]. Therefore, reliable data on abundance is needed to assess the status of all franciscana population and to establish proper conservation actions. In this sense, the use of the availability correction factors presented here should be encouraged to correct for availability bias of fransicana in aerial surveys and, consequently, obtain more realistic estimates of the impact of bycatch and other human activities to this endangered species.
